# *In situ* estimation of cotton fourth internode length and height-to-node ratio using UAV-derived vegetation indices and machine learning algorithms

**DOI:** 10.3389/fpls.2025.1722440

**Published:** 2025-12-16

**Authors:** Peter C. Ngimbwa, Denis O. Kiobia, Canicius J. Mwitta, Wesley M. Porter, Javad M. Velni, Glen C. Rains

**Affiliations:** 1 College of Engineering, University of Georgia, Tifton, GA, United States; 2 Department of Entomology, University of Georgia, Tifton, GA, United States; 3 Department of Crop and Soil Sciences, University of Georgia, Tifton, GA, United States; 4 Department of Mechanical Engineering, Clemson University, Clemson, SC, United States

**Keywords:** crop phenotyping, decision support, plant growth regulator, precision agriculture, remote sensing

## Abstract

This study investigates the potential of utilizing nonparametric, nonlinear machine learning (ML) algorithms, in conjunction with vegetation indices (VIs) derived from unmanned aerial vehicles (UAVs), to estimate the height-to-node ratio and the fourth internode length in cotton plants. The objective was to enhance the monitoring of these traits, thereby providing more accurate guidance on the optimal timing of plant growth regulator (PGR) applications. Data was collected from eight plots in our experimental field, with six plots used for model training and two for testing. During model development, the performance was assessed using nested 5-fold cross-validation, repeated three times with different partitions. For each algorithm, hyperparameters were tuned on the inner folds via Bayesian optimization with a Gaussian process surrogate, and the tuned model was evaluated on the corresponding outer test fold. We evaluated the performance of the ML algorithms using the Friedman test and interpreted their result using the Wilcoxon signed-rank test. The results demonstrate that VIs, combined with ML algorithms, can reliably estimate both the height-to-node ratio and the length of the fourth internode. Additionally, among the tested ML algorithms, Support Vector Regression (SVR) demonstrated superior performance for predicting height-to-node ratio, with an R² value of 0.8257 (95% CI: 0.7404 - 0.9110), RMSE value of 0.0998 (95% CI: 0.0953 - 0.1044), and rRMSE value of 5.51 (95% CI: 5.30 - 5.7). Meanwhile, the CatBoost demonstrated higher performance in estimating the fourth internode length, with an R² value of 0.799 (95% CI: 0.7570 - 0.8415), an RMSE of 0.1788 (95% CI: 0.1631 - 0.1945), and a rRMSE of 10.64 (95% CI: 9.90 - 11.38). Furthermore, using the Shapley Additive exPlanations (SHAP) approach, we revealed the contribution of each of the VI to the model’s prediction. Overall, the findings demonstrate that UAV-derived VIs, combined with a machine learning algorithm, can consistently estimate these cotton traits. Additionally, this approach can replace traditional field-based measurements, thereby supporting more efficient monitoring and precise PGR management decisions.

## 1 Introduction

Since their introduction in the early 1980s, plant growth regulators (PGRs) have become essential for the effective production of cotton (*Gossypium hirsutum L*.). These hormone products function by controlling the growth rate, specifically by reducing internode elongation, limiting plant height, and preventing excessive vegetative growth (Nuti et al., 2006). They achieve this by partially inhibiting the gibberellic acid hormone, which limits cell wall loosening and restricts cell elongation, leading to a shorter and more compact canopy structure. This compactness enhances fruit and boll retention (Cothren and Oosterhuis, 2010). It also reduces the risk of boll rot and fungal diseases by improving the penetration of insecticides and fungicides into plants. Additionally, a more compact structure enables more efficient operation of mechanical equipment, such as cotton harvesters (Vaz et al., 2023). In some cases, the use of PGRs can lead to higher yields (Biles and Cothren, 2001; Leal et al., 2020) and improved cotton fiber quality (Mao et al., 2015; Chalise et al., 2022).

Despite their benefits, the effectiveness of PGRs in balancing vegetative and reproductive growth is heavily dependent on timing. Applying PGRs too early may lead to early cutout, while applying them too late may fail to sufficiently restrict the plant’s growth (Hand et al., 2022). Both scenarios can ultimately affect the yield and fiber quality (Chalise et al., 2022). For this reason, farmers monitor their fields so they can apply PGR at the right time. The two common methods used to determine the appropriate application time are: measuring the cotton plant height-to-node ratio and measuring the fourth internode length (Hand et al., 2022). The fourth internode length is an area of active elongation on the main cotton stem, reflecting the plant’s growth potential. It is determined by measuring the length between the fourth and fifth nodes on the main stem from the top of the plant. The height-to-node ratio is calculated by dividing the plant’s height by the total number of nodes. This ratio provides insight into the vigor of the cotton plant’s vegetative growth (Kerby et al., 1997) and guides the aggressiveness of the PGR program. This includes both the PGR application rate and follow-ups to manage excessive vegetation growth. Typically, a PGR application is considered when the fourth internode length exceeds 5 cm, and when the height-to-node ratio at early squaring, first bloom, and early bloom exceeds 1.3, 1.9, and 2.5, respectively (Hand et al., 2022).

Traditionally, these measurements are taken manually on a weekly basis using a tape measure or a ruler on 20 randomly selected plants across the field. These measurements are then averaged to provide a representative value for the entire field (Hand et al., 2022). This process can be labor-intensive, time-consuming, and prone to error, making it less likely to accurately reflect growth variations across the field. Failing to detect variations in growth leads to over-application of PGRs and may further increase variability in plant growth and yield (Trevisan et al., 2018). Additionally, as the cotton plants grow and the canopy thickens, collecting data by walking through the field can cause damage to the plants (Wu et al., 2022).

To address these challenges, a variety of innovative technological solutions have been proposed, including Light Detection and Ranging (LiDAR), ground-based sensors, and unmanned aircraft vehicles (UAVs) equipped with remote sensing technology. For instance, Mccarthy et al. (2010) developed a ground-sensing system that mounted a vision system on an in-field vehicle to detect and measure the fourth internode length, achieving an accuracy ranging from 12% to 64%. Its effectiveness was constrained by the visual occlusion of the main stem nodes by foliage and the angle of sunlight in relation to the camera’s view. Sun et al. (2021) employed a high-resolution terrestrial LiDAR system combined with the skeletonization algorithm to estimate the length of the main cotton stem and the number of nodes, achieving an average R^2^ of 94% and 70%, respectively. More recently, Saeed et al. (2023) extended this line of research by developing a 3D data annotation tool that utilizes deep learning techniques to enhance the extraction of cotton traits, such as main stem length and the number of nodes. Their approach successfully estimated these parameters, yielding an R² value above 80%. Both Saeed et al. (2023) and Sun et al. (2021) methods can be applied to estimate the height-to-node ratio and indirectly estimate the fourth internode length. Overall, these methods underscore the practicability of using ground sensing systems to directly or indirectly estimate the height-to-node ratio and fourth internode length. However, it is important to note that a ground-sensing system can be destructive as the crop grows and takes time to traverse an entire field (Xu et al., 2019).

Given these constraints, there has been a growing shift towards using unmanned aerial vehicles (UAVs) for data acquisition. UAVs provide rapid, and non-destructive coverage of large areas, enabling frequent data collection using vegetation indices (VIs). Vegetation indices are spectral indicators derived mathematically from reflectance values in visible and near-infrared regions of the electromagnetic spectrum. These indices are utilized to assess plant vigor, growth, and various biophysical characteristics (Xue and Su, 2017). Traditionally, researchers have used parametric regression methods to assess plant biophysical and phenotypic attributes in relation to VIs (Souza et al., 2017; Lacerda et al., 2022). While parametric regression methods are simple and interpretable, they are often constrained by their assumptions regarding an explicit relationship between VIs and plant attributes. These limitations can lead to inadequate modeling of more complex relationships (Ma et al., 2022). To address these challenges, recent studies have employed nonparametric and nonlinear regression methods, including machine learning (ML) algorithms, to model these relationships more accurately and improve the estimation of cotton biophysical and phenotypic attributes. ML algorithms, which are a subfield of artificial intelligence, are particularly effective at capturing nonlinear relationships and detecting complex patterns. Unlike the parametric methods, they do not require a predefined functional form or assumptions about the data’s distribution. Instead, these algorithms learn the relationship between the explanatory variables and the response variable without prespecifying a functional form (Imam et al., 2024). This capability enables them to capture complex nonlinear interactions between spectral signatures and plant architecture that parametric models might miss, thereby improving prediction accuracy. This capability is evident in studies that estimate leaf area index (Ma et al., 2021; Lang et al., 2023), cotton yield (Ma et al., 2022; Lang et al., 2023), and cotton height (da Silva Andrea et al., 2023; Psiroukis et al., 2023).

Despite advances in using ML and VIs to estimate broader cotton parameters, research on estimating fourth internode length and the height-to-node ratio using these methods remains unexplored. Therefore, this study aims to address this research gap by evaluating whether vegetation indices, coupled with machine learning algorithms, can accurately predict fourth internode length and the height-to-node ratio. Specifically, it seeks to: (i) validate the effectiveness of using vegetation indices to estimate fourth internode length and heighttonode ratio; (ii) identify the most influential VIs for the prediction of the mentioned cotton traits using Shapley Additive Explanations (SHAP); and (iii) identify the most effective machine learning model(s) for estimating these cotton traits. The findings of this study will facilitate the rapid estimation of both the fourth internode length and the height-to-node ratio, thereby optimizing the timing of PGR application in cotton fields.

## 2 Materials and methods

### 2.1 Study area

**Figure 1:**
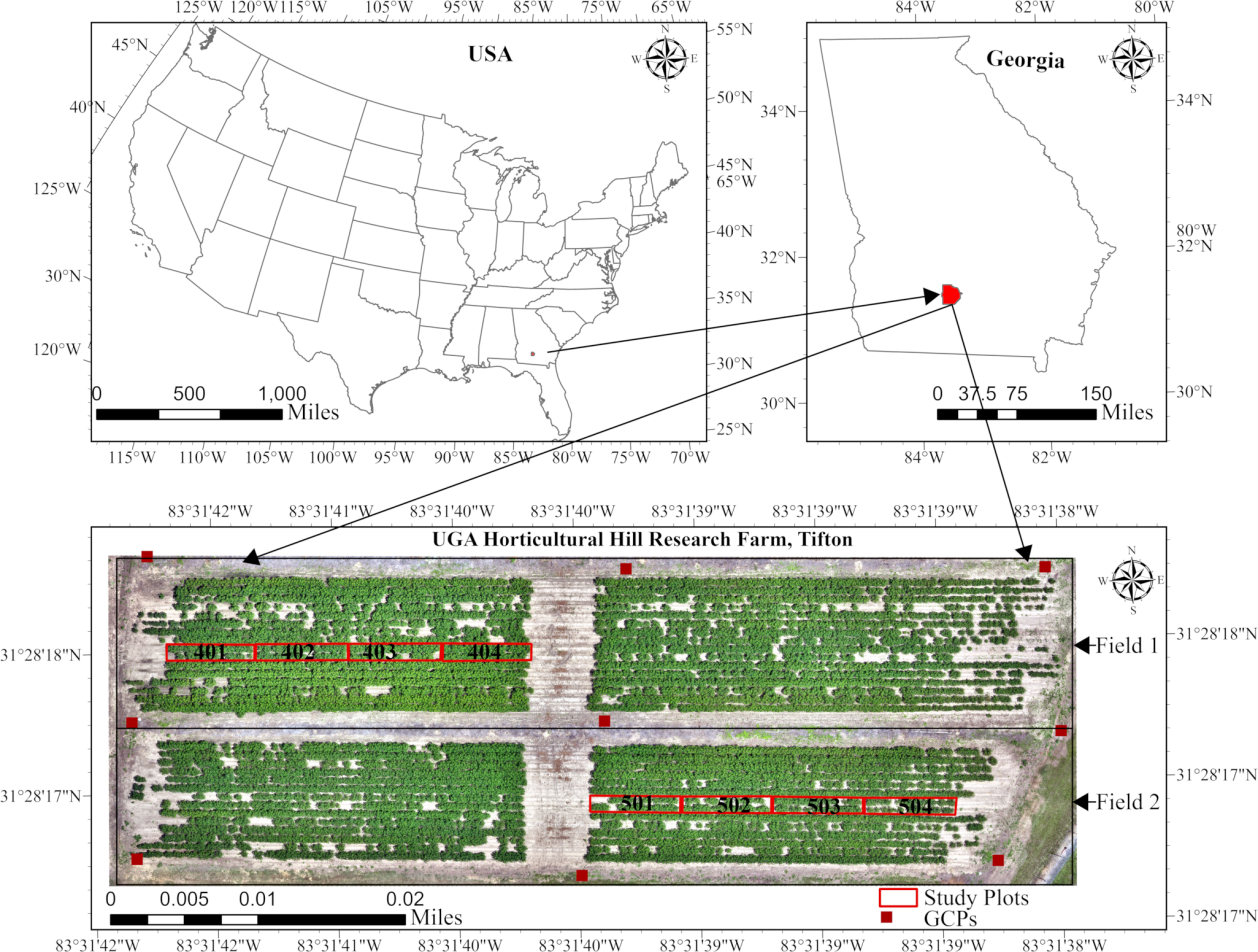
Field layout with the marked monitored plot for the 2025 season in Tifton, GA. USA.

The study was conducted during the 2025 season at the Horticultural Hill Research Farm, located on the University of Georgia’s Tifton Campus in Tifton, Georgia. The farm is situated at latitude 31°28′18″N and longitude 83°31′39″W. The farm comprises two cotton fields, each with 24 plots. Data collection and monitoring focused on four specific plots per field, as shown in **Figure 1**. The cotton cultivar used in this study was PHY360W3FE (PhytoGen cottonseed), which was planted on May 9th, 2025, at a seeding rate of 29,000 seeds per acre. The seeds were sown at a depth of 1.9cm, with an in-row spacing of 10.2cm.

The experiment followed a split-plot design, with irrigation (irrigated and non-irrigated) as a whole-plot factor applied at the field level and PGR application (applied vs. non-applied) as a subplot factor applied at the plot level. Within each field, plots served as independent replicates. Field 1 was designated as the irrigated field, while Field 2 was the non-irrigated field. Field 1 received weekly irrigation based on the cotton water requirements established by the University of Georgia Extension (Hand et al., 2023). Irrigation was used as a treatment factor because it significantly influences excessive vegetation growth in cotton plants (Hand et al., 2022). The PGR, Mepiquat Chloride 4.2, was applied at the rate of 16oz/acre to plots 401 and 402 in Field 1 and to plots 503 and 504 in Field 2 on two occasions: July 2 and July 21, 2025. Each plot measured 914cm in length, 91cm wide, and was spaced 91cm apart.

### 2.2 Ground data acquisition

**Figure 2:**
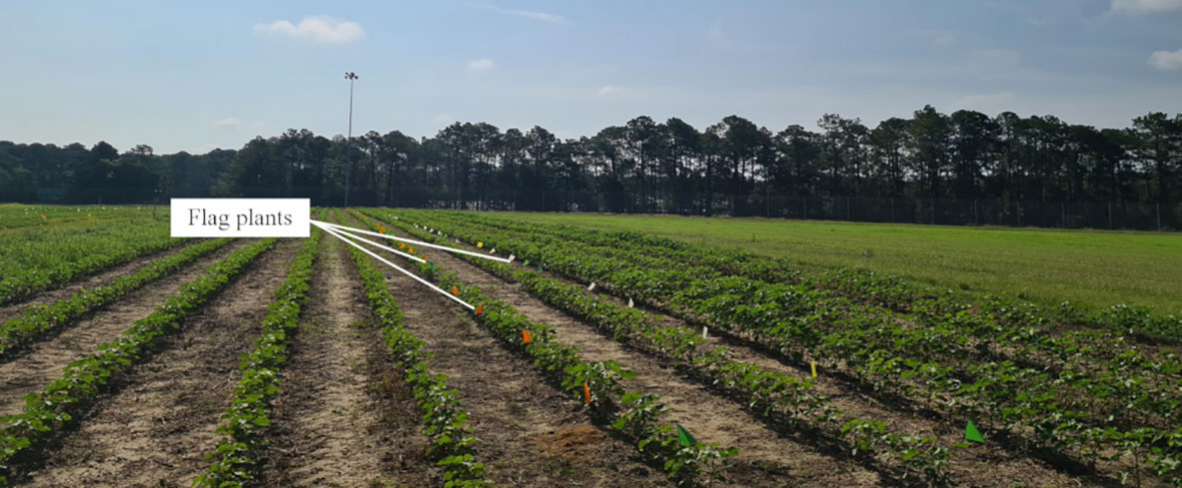
Plastic field flag used to mark the cotton plants for repeated measurements. Flags were placed adjacent to the target stem. The same flagged plants were revisited on each measurement date throughout the season.

At the start of the season, right after plant emergence, eight cotton plants were selected for the data collection in each monitored plot. Each plant was individually marked with a plastic field flag, positioned next to the plant to avoid contact with the main stem (**Figure 2**). These flags remained in place throughout the study, allowing for accurate identification of the monitored plants on each data collection date. On each scheduled measurement date, the number of nodes, height, and the fourth internode length of the marked plants were recorded. The height of the plant and the length of the fourth internode were measured using a ruler, and the readings were taken to the nearest 0.64 cm. The measurements were taken twice a week from June 5 to August 22, covering all critical stages of cotton growth.

### 2.3 UAV imagery acquisition

We collected UAV imagery with a DJI Mavic 3 Multispectral UAV on the same day as the ground measurements, immediately afterward. The DJI Mavic 3 Multispectral is equipped with dual sensor systems: an RGB sensor capturing visible red (R), green (G), and blue (B) wavelengths, and a multispectral sensor recording both visible (green: 560 ± 16 nm, red: 650 ± 16 nm) and non-visible (red edge (RE): 730 ± 16 nm, near-infrared (NIR): 860 ± 26 nm) wavelengths. Before each flight, we used a MicaSense calibrated reflectance panel (serial ID RP04-1815010-SC) to obtain reference reflectance values for the radiometric calibration of the images acquired by the multispectral sensors. The manufacturer-certified reflectance values for this panel are as follows: 50.6% (475–20 nm) for Blue, 50.9% (560–20 nm) for Green, 51.1% (668–10 nm) for Red, 51.1% (717–10 nm) for Red Edge, and 51.2% (840–40 nm) for NIR. The UAV was flown autonomously along the predefined mapping path at an average horizontal speed of 3.5 km/h and at a course angle of 271°. This angle was set to minimize turns and maintain consistent overlap across the study area. The flights were scheduled between 11:00 a.m. and 2:00 p.m. to minimize light variability in the data by targeting a high local solar elevation angle, thereby ensuring consistent illumination and minimal shadowing effect (Wu et al., 2022; Wang et al., 2025). Images were captured at 3-second intervals, with 70% side overlap and 80% forward overlap. The RGB images had a resolution of 5280 × 3956 pixels, while the multispectral images had a resolution of 2592 × 1944 pixels. The flight altitude was 20 meters, and the number of images captured during each flight was 273. The resulting orthomosaic imagery had a ground sampling distance (GSD) of approximately 0.93 cm per pixel. An Emlid Reach RS+ GNSS receiver was used as a base station to provide real-time kinematic (RTK) corrections, improving the position accuracy of GNSS data collected by the UAV. This enhancement was crucial for generating reliable and spatially accurate imagery.

### 2.4 Data pre-processing

Following the UAV flight, all images were downloaded and processed using Pix4Dmapper software (v4.9.0; Pix4D SA, Lausanne, Switzerland) on a desktop computer running a 64-bit Windows operating system (x64-based processor). The computer was equipped with an Intel Core i9-14900K CPU at 3.20 GHz with 24 Cores, 64 GB of RAM, and an NVIDIA GeForce RTX 4090 GPU with 34 GB of dedicated memory. The processing involved three steps: (1) extraction and matching of common characteristics points (keypoints) from the images to calibrate the internal and external parameters of the camera; (2) generating point cloud and mesh using default settings (minimum image matches=3, image scale=0.4, matching window=7x7pixels); and (3) creation of georectified RGB and multispectral orthomosaics, along with multispectral vegetation indices. Radiometric calibration for multispectral data used the “Camera and Sun Irradiance” setting to correct reflectance values. Furthermore, to enhance georeferencing accuracy, nine GCPs were added and marked using the rayCloud interface, with manual marking applied to four images per GCP, and the remaining images were automatically marked using Pix4Dmapper’s Automatic Marking tool. Final orthomosaics and vegetation indices were exported as georeferenced TIFF files in the WGS 1984 UTM Zone 17N projected coordinate system (EPSG:32617).

Mean spectral reflectance values for each plot were extracted from the multispectral orthomosaics using ArcGIS Pro (v3.3; ESRI, Redlands, CA, USA). To isolate the canopy from the soil, the excess green (ExG) vegetation index (Woebbecke et al., 1995) was derived from the RGB orthomosaics. An ExG threshold value of 0.2 was then applied to separate the canopy from the soil. This threshold was determined through human visual inspection of the canopy in the RGB images. For the ground data, we calculated the average measurements from eight marked plants per plot on each data collection date to determine the mean node count, plant height, and the fourth internode length. The height-to-node ratio was computed by dividing the mean height by the mean node count, as shown in the formula below (Equation 1). The total sample size across all plots and dates was 136.

(1) $$height-to-node\;ratio=\frac{Average\;Plant\;height}{Average\;node\;count}$$

### 2.5 Vegetation features

Vegetation indices chosen for this study are listed in **Table 1**. We selected these indices based on their demonstrated effectiveness in quantifying in-season plant growth parameters (see references in the table). Additionally, they are compatible with the spectral bands accessible on our UAV platform.

**Table 1:** Vegetation indices derived from multispectral and RGB imagery.

Abbreviations	Full name	Formula	Reference
Multispectral Vis			
NDVI	Normalized Difference Vegetation Index	$\left(\frac{NIR-R}{NIR+R}\right)$	(Rouse et al., 1973)
NDRE	Normalized Difference Red-Edge Index	$\left(\frac{NIR-RE}{NIR+RE}\right)$	(Barnes et al., 2000)
OSAVI	Optimized Soil-Adjusted Vegetation Index	$\left(\frac{NIR-R}{NIR+R+0.16}\right)$	(Rondeaux et al., 1996)
GNDVI	Green Normalized Difference Vegetation Index	$\left(\frac{NIR-G}{NIR+G}\right)$	(Gitelson et al., 1996)
NLI	Non-Linear Index	$\left(\frac{NIR^{2}-R}{NIR^{2}+R}\right)$	(Goel and Qin, 1994)
EVI2	Enhanced Vegetation Index 2	$2.5\cdot \left(\frac{NIR-R}{NIR+2.4\cdot R+1}\right)$	(Jiang et al., 2008)
GCI	Green Chlorophyll Index	$\left(\frac{NIR}{G}\right)-1$	(Gitelson et al., 2003)
MCARI	Modified Chlorophyll Absorption Ratio Index	$\left(\left(NIR-R\right)-0.2\cdot \left(NIR-G\right)\right)\cdot \left(\frac{NIR}{R}\right)$	(Daughtry et al., 2000)
RDVI	Renormalized Difference Vegetation Index	$\frac{\left(NIR-R\right)}{\sqrt{NIR+R}}$	(Roujean and Breon, 1995)
MSR	Modified Simple Ratio	$\frac{\left(\frac{NIR}{R}\right)-1}{\sqrt{\left(\frac{NIR}{R}\right)+1}}$	(Chen, 1996)
RGB Vis			
NGRDI	Normalized green-red difference index	$\frac{\left(g-r\right)}{\left(g+r\right)}$	(Hunt et al., 2005)
ExG	Excessive green index	$\frac{\left(2\cdot g-b-r\right)}{\left(b+g+r\right)}$	(Woebbecke et al., 1995)
GCC	Green Chromatic Coordinates	$\frac{g}{\left(r+g+b\right)}$	(Woebbecke et al., 1995)

### 2.6 Description of the machine learning models

In this study, we evaluated seven nonparametric, nonlinear regression algorithms for predicting the fourth internode length and the height-to-node ratio from VIs. The algorithms evaluated include Random Forests (RF), Support Vector Regression (SVR), Decision Trees (DT), Categorical Boosting (CatBoost), Light Gradient Boosting Machine (LGBM), Extreme Gradient Boosting (XGBoost), and Gradient Boosting (GB). We incorporated this diverse set of algorithms to conduct a comprehensive comparison across distinct modeling paradigms and identify the most accurate and generalized model for these cotton traits, as outlined in the subsequent section 2.8.

SVR, a regression variant of the Support Vector Machine (SVM), uses ϵ-insensitive loss and kernel functions, such as linear, polynomial (Poly), radial basis function (RBF), and sigmoid to model smooth, nonlinear relationships (Schölkopf and Smola, 2002), making it robust for modeling complex, nonlinear relationships between VIs and cotton traits. DT recursively partitions the feature space using a greedy algorithm that, at each node, selects the split that minimizes the sum of squares error for a regression task. This process continues until certain stopping rules, such as maximum depth and minimum samples, are met (Hastie et al., 2009). This structure offers a flexible baseline for capturing nonlinear relationships in the morphological traits of cotton derived from Vegetation Indices (VIs). Random Forests (RF) extend DT by combining many bootstrapped trees using bagging, in which multiple bootstrap samples are drawn from the original data, and separate trees are trained independently on each sample. By averaging the predictions from these weakly correlated trees, RF reduces variance and overfitting (Liaw and Wiener, 2002), thus enhancing robustness against collinearity among vegetation indices in data obtained from UAVs. Boosting-based methods (GB, XGBoost, LGBM, and CatBoost) iteratively add shallow trees that correct the errors made by previous trees. This approach allows them to model complex, nonlinear relationships between vegetation indices and cotton traits while leveraging regularization and efficient training to achieve improved predictive accuracy. GB provides a foundational form of gradient boosting (Friedman, 2001). XGBoost builds upon GB by adding extra regularization and exploiting parallelization, thereby improving generalization and training speed (Chen and Guestrin, 2016). LGBM employs leaf-wise (best-first) tree growth, histogram-based splitting, gradient-based sampling, and feature bundling to handle large feature sets efficiently and improve both learning speed and predictive accuracy (Ke et al., 2017). CatBoost uses ordered boosting to address prediction shift caused by target leakage and symmetric (oblivious) trees to help stabilize training and reduce overfitting, while making inferences quickly (Prokhorenkova et al., 2018).

### 2.7 Evaluation metrics

This study employed the coefficient of determination (R²), root mean square error (RMSE), and relative root mean square error (rRMSE)to evaluate the accuracy of the models. The R² (Equation 2) value measures the proportion of variation in the dependent variables that the model can explain. The R^2^ value ranges from 0 to 1, where 1 indicates that the model explains all variation in the data, and 0 indicates that it has no explanatory power. RMSE (Equation 3) and rRMSE (Equation 4) measure the prediction error of the model by quantifying the differences between the predicted and the actual values. For both RMSE and rRMSE, smaller values indicate more accurate predictions, while larger values suggest poorer model accuracy. The formulas for calculating R^2^, RMSE, and rRMSE are presented below.

(2) $$R^{2}=1-\frac{\sum_{i=1}^{n}{\left(y_{i}-{\hat{y}}_{i}\right)}^{2}}{\sum_{i=1}^{n}{\left(y_{i}-\bar{y}\right)}^{2}}$$

(3) $$RMSE=\;\sqrt{\frac{1}{n}\sum_{i=1}^{n}{\left(y_{i}-{\hat{y}}_{i}\right)}^{2}}$$

(4) $$rRMSE=\;\frac{RMSE}{\bar{y}}\;\times \;100$$

Where $y_{i}$ represent the actual measured value, $\bar{y}$ denotes the mean of the observed values, and ${\hat{y}}_{i}$ is the value predicted by the model.

### 2.8 Model training and evaluation

**Figure 3:**
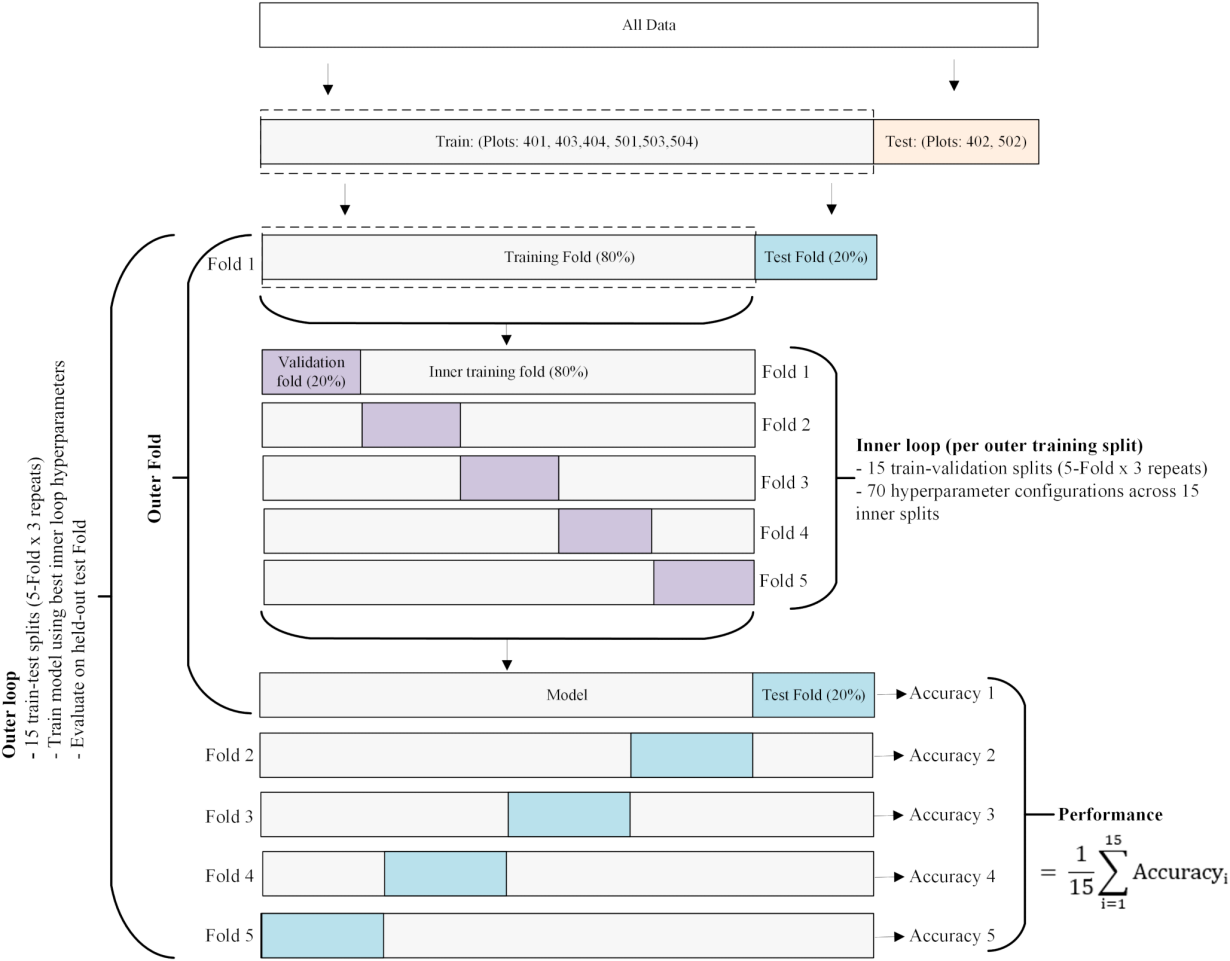
The workflow of the nested cross-validation procedure for model training, hyperparameter tuning, and model validation. Data from six plots (401, 403, 404, 501, 503, and 504) were used for nested cross-validation. The data from plots 402 and 502 were reserved as an independent test set for the top-ranked algorithm, selected via statistical testing. In the outer loops, the models were trained on the full training set using the optimal hyperparameters identified in the inner loop. These hyperparameters were determined by evaluating 70 configurations via Bayesian optimization in the inner loop.

Two of the eight experimental plots were randomly selected and reserved for model testing. One was drawn from an irrigated field and the other from a non-irrigated field. The remaining six plots were used for model development. Within the model development phase, we trained and evaluated the performance of the machine learning algorithms using a nested cross-validation approach. This nested structure, illustrated in **Figure 3**, was adopted because it provides an unbiased estimate of the model’s generalization error under tuned parameters, thereby reducing the risks of model bias, overfitting, and underfitting (Cawley and Talbot, 2010). In the outer loop, we performed 5-fold cross-validation, repeated 3 times, resulting in 15 independent train-test splits. For each iteration, 20% of the data was held as the validation set, while the remaining 80% was used for model training. Using the outer training partition, we performed an inner 5-fold cross-validation repeated three times for hyperparameter optimization, yielding 15 train-validation splits. Within the inner loop, 80% of the data was allocated for model fitting and 20% for validation. The hyperparameter tuning was conducted using Bayesian optimization with a Gaussian process technique (Williams and Rasmussen, 2006) for 70 iterations. This resulted in 1050 models fitting per outer split (70 x 15). During hyperparameter tuning, rRMSE was used as the primary criterion for selecting the best hyperparameter configurations for retraining the model in the outer loop. If two models had the same optimal rRMSE, R² served as the tiebreaker. The final performance was assessed on the corresponding outer test fold, resulting in 15 performance estimates per algorithm. These performance estimates were subsequently compared across models to determine the best-performing algorithm using the Friedman test (Demšar, 2006). We applied the Friedman test ( ${\text{χ}}_{F}^{2}$) to test H_0_: no significant difference in predictive performance among the ML algorithms vs H_1_: at least one of the ML algorithms performed significantly better than the others. If the Friedman test indicated a significant result, a *post-hoc* test was conducted using the Wilcoxon signed-rank test (Wilcoxon, 1945) to interpret the result. Additionally, we used the Shapley Additive exPlanation (SHAP) technique to interpret the contribution of each variable to the model. SHAP uses a game theory approach to compute the Shapley value, quantifying the contribution of each predictor variable to the model (Lundberg and Lee, 2017). To ensure reproducibility, a fixed random seed (RANDOM_STATE = 42) was used across all runs. All analyses were conducted in Python 3.12 within a Jupyter Notebook environment. The description of the hyperparameters used for hyperparameter tuning is shown in **Supplementary Table 1**.

## 3 Results

### 3.1 Growth variation of the fourth internode length and height-to-node ratio

**Figure 4:**
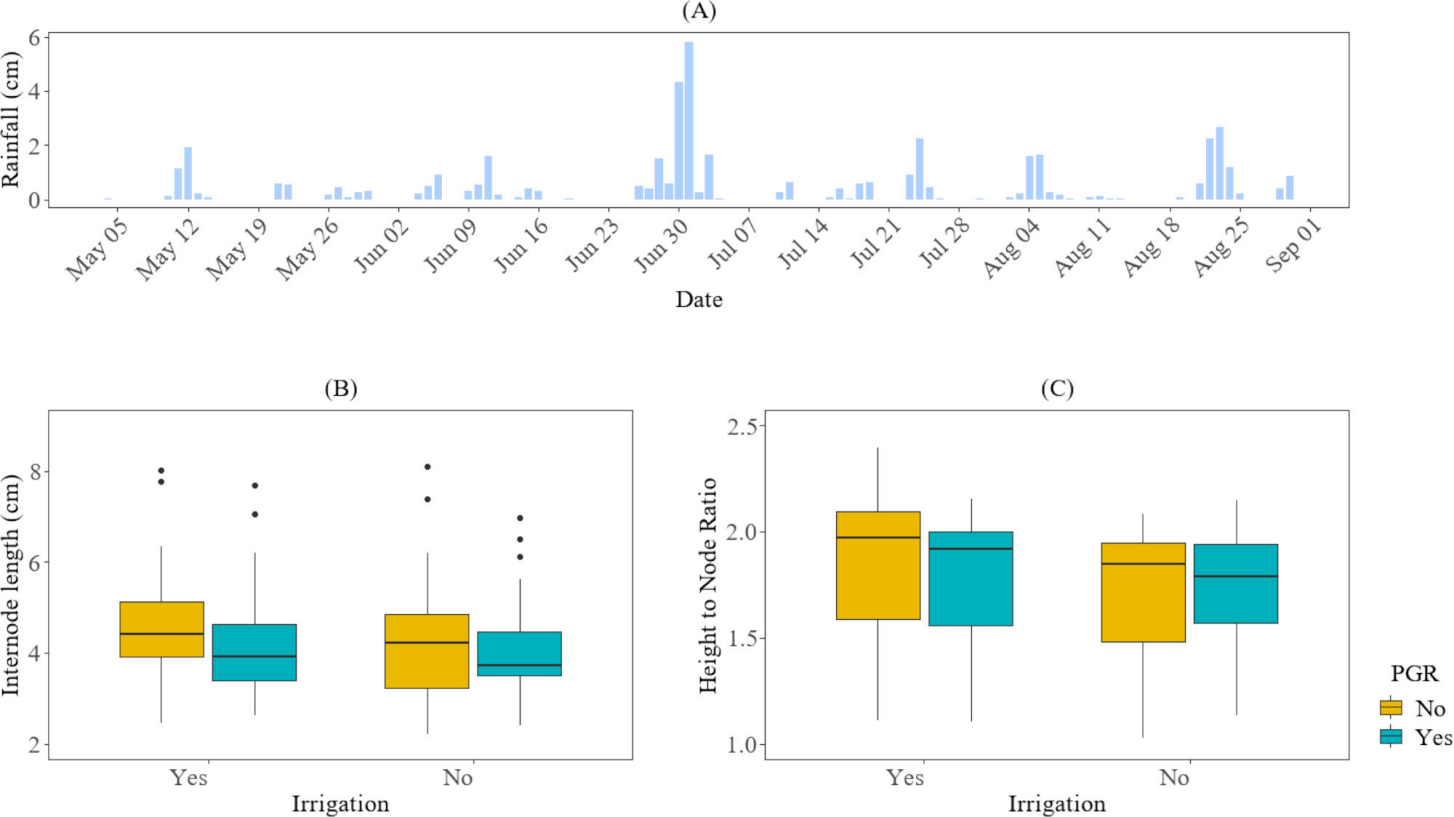
The impact of irrigation and PGR application on cotton growth in the 2025 season at HortHill farm. **(A)** illustrates the amount of rainfall received at HortHill from May to August 2025. **(B)** depicts the effect of PGR and Irrigation on the variation of the fourth internode length, with the measurement pooled across all sampling dates to summarize the season distribution of this trait. **(C)** demonstrates the corresponding effect of PGR and Irrigation on the growth variation for the cotton plant height-to-node ratio, also based on data aggregated across the entire monitoring period to summarize its seasonal distribution.

On average, across both irrigation levels, the plants in the plots without PGR applications (plots 403–404 in field 1 and plots 501–502 in field 2) exhibited longer fourth internode lengths and a higher height-to-node ratio compared to the plots that received PGR applications (in the plots with PGR applications (plots 401–402 in field 1 and plots 503–505 in field 2), as shown in **Figure 4**. However, these differences were not statistically significant (P-value > 0.05). This lack of significance could be attributed to the amount of rainfall received during the 2025 season. Typically, excessive rainfall is a major contributor to increased vegetative growth in cotton.

**Figure 5:**
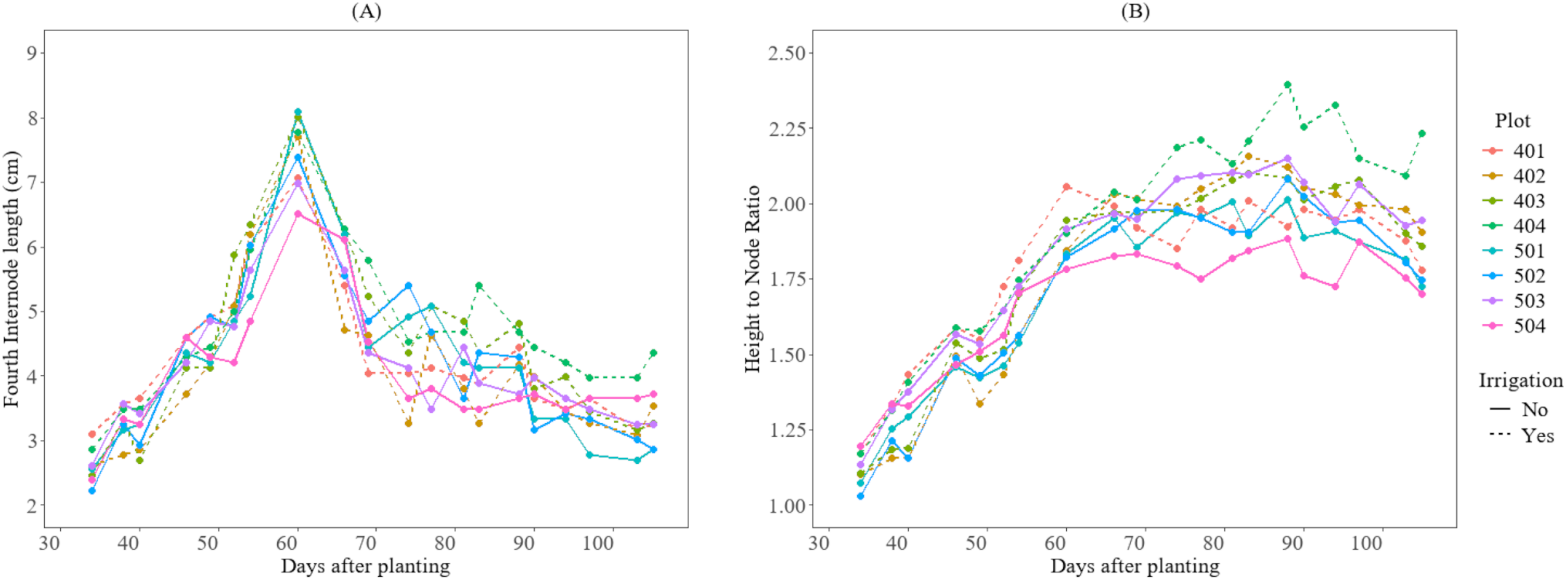
Growth variations of the cotton crops after planting day for the 2025 season at HortHill farm. **(A)** illustrates the variation of the length of the fourth internode over time, and **(B)** shows the growth variation of the height-to-node ratio over time.

Furthermore, as illustrated in **Figure 5**, although there was no significant difference in growth between treatments, plots under irrigated conditions reached approximately 5 cm in the fourth internode length approximately 52 days after planting. In contrast, the non-irrigated plots reached the same internode length 54 days after planting. Additionally, internode elongation in both fields reached its peak length around 60 days after planting (DAP). For the height-to-node ratio, the irrigated plots reached 1.3 at 38 DAP and 1.9 at 60 DAP, while non-irrigated plots reached 1.3 at 40 DAP and 1.9 at 80 DAP.

### 3.2 Cross-validation performance of the models for the fourth internode length prediction

**Table 2** presents the cross-validation performance of the SVR, GB, CatBoost, RF, LGBM, XGBoost, and DT for predicting the fourth internode length. The CatBoost algorithm achieved the best performance, with the lowest rRMSE value of 10.64, with a 95% confidence interval (CI) ranging from 9.90 to 11.38. It also achieved the highest coefficient of determination (R^2^) of 0.799, with a 95% CI of 0.757 to 0.8415. RF and SVR follow closely behind, with rRMSE values of 10.81 and 10.88, and R^2^ values of 0.795 and 0.783. GB, XGBoost, and LGBM exhibited slightly weaker performance compared to CatBoost, with rRMSE and R^2^ values ranging from 11.17 to 11.52 and 0.779 to 0.744, respectively. Conversely, DT achieved an rRMSE value of 15.15 and an R² of 0.559, indicating a higher predictive error and limited ability to capture the complex, nonlinear relationship in the data.

**Table 2:** Cross-validation performance (mean values with 95% CI) of models for predicting the fourth internode length of cotton plants using VIs.

Model	rRMSE (95% CI)	R^2^ (95% CI)	RMSE (95% CI)
CatBoost	10.64 (9.90, 11.38)	0.7993 (0.7570, 0.8415)	0.1788 (0.1631, 0.1945)
RF	10.81 (9.91, 11.71)	0.7950 (0.7502, 0.8399)	0.1819 (0.1636, 0.2003)
SVR	10.88 (10.06, 11.70)	0.7830 (0.7229, 0.8431)	0.1825 (0.1676, 0.1974)
GB	11.17 (10.43, 11.91)	0.7787 (0.7322, 0.8253)	0.1876 (0.1720, 0.2032)
XGBoost	11.26 (10.44, 12.07)	0.7741 (0.7211, 0.8271)	0.1892 (0.1728, 0.2056)
LGBM	11.52 (9.86, 13.19)	0.7443 (0.6493, 0.8394)	0.1935 (0.1637, 0.2234)
DT	0.1515 (0.1324, 0.1706)	0.5590 (0.3895, 0.7285)	0.2536 (0.2210, 0.2862)

### 3.3 Cross-validation performance of the models for the height-to-node ratio prediction

**Table 3** illustrates the cross-validation performance of SVR, GB, CatBoost, RF, LGBM, XGBoost, and DT for predicting the cotton height-to-node ratio. The SVR algorithm achieved the highest accuracy, with an rRMSE of 5.51, a 95% CI of 5.3 to 5.72, and an R² of 0.8257 with a 95% CI of 0.7404 to 0.9110. In comparison, the ensemble tree-based algorithms (CatBoost, GB, RF, LGBM, XGBoost) had an rRMSE ranging from 5.83 to 6.99, and an R^2^ value between 0.7512 and 0.8202. This suggests moderate predictive power compared to that of SVR. On the other hand, DT achieved the lowest predictive performance, with an rRMSE value of 8.45 and an R^2^ value of 0.621.

**Table 3:** Cross-validation performance (mean values with 95% CI) of models for predicting the height-to-node ratio of cotton plants using VIs.

Model	rRMSE (95% CI)	R^2^ (95% CI)	RMSE (95% CI)
SVR	5.51 (5.30, 5.72)	0.8257 (0.7404, 0.9110)	0.0998 (0.0953, 0.1044)
CatBoost	5.83 (5.43, 6.22)	0.8202 (0.7532, 0.8871)	0.1054 (0.0988, 0.1121)
GB	6.20 (5.82, 6.59)	0.8000 (0.7353, 0.8647)	0.1123 (0.1057, 0.1189)
RF	6.36 (5.82, 6.91)	0.7822 (0.7098, 0.8546)	0.1154 (0.1050, 0.1258)
LGBM	6.47 (5.94, 7.00)	0.7661 (0.6544, 0.8778)	0.1170 (0.1082, 0.1258)
XGBoost	6.99 (6.46, 7.52)	0.7512 (0.6768, 0.8256)	0.1265 (0.1178, 0.1352)
DT	8.45 (7.45, 9.45)	0.6210 (0.4635, 0.7785)	0.1527 (0.1352, 0.1703)

### 3.4 Statistical comparison of the models’ performance

The Friedman test based on rRMSE from 15 outer cross-validation folds for each ML algorithm indicated that at least one of the ML algorithms performed significantly better than the others in predicting the fourth internode length ( ${\text{χ}}_{F}^{2}$ = 30.457, p-value< 0.05). Following Friedman test, we ranked the models by computing The CatBoost (average rank = 2.8) was ranked first, followed by RF (average rank = 2.8667), SVR (average rank = 3.40), LGBM (average rank = 3.60), GB (average rank = 4.3333), XGBoost (average rank = 4.60), and DT (average rank = 6.40) as shown in **Table 4**. In addition, when we performed *post-hoc* tests using Wilcoxon signed-rank tests with the Holm adjustment to compare other models to the first-ranked model, the results indicated that the CatBoost significantly outperformed the GB (p-value = 0.0008), the DT (p-value = 0.0004), and the XGBoost models (p-value = 0.0205). No significant difference was observed in the estimates between CatBoost and RF (p-value = 0.7197), SVR (p-value = 0.7197), and LGBM (p-value = 0.3786). However, since the CatBoost model’s mean rRMSE and R² exceed those of the other models, it was selected as the best model for predicting the fourth internode length.

**Table 4:** *Post-hoc* results following the Friedman test, showing the average rank and Holm-adjusted one-sided Wilcoxon p-value of the models for predicting the fourth internode length. The model with the lower average rank value is the better model for prediction.

Model	Average ranks value	Comparison vs CatBoost	Holm-adjusted p-value
CatBoost	2.8000	–	–
RF	2.8667	CatBoost vs RF	0.7197
SVR	3.4000	CatBoost vs SVR	0.7197
LGBM	3.6000	CatBoost vs LGBM	0.3786
GB	4.3333	CatBoost vs GB	0.0008
XGBoost	4.6000	CatBoost vs XGBoost	0.0205
DT	6.4000	CatBoost vs DT	0.0004

Similarly, the Friedman test based on rRMSE for predicting the height-to-node ratio has also indicated that at least one of the ML algorithms performed significantly better than the others ( ${\text{χ}}_{F}^{2}$ = 48.143, p-value< 0.05). The SVR (average rank = 2.2) was ranked first, followed by the CatBoost (average rank = 2.2667), the GB (average rank = 3.5333), the RF (average rank = 3.8000), the LGBM (average rank = 4.3333), the XGBoost (average rank = 5.2667), and the DT (average rank = 6.6000) as shown in **Table 5**. The *post-hoc* tests using Wilcoxon signed-rank tests with the Holm adjustment to compare other models to the first-ranked model indicated that the SVR significantly outperformed the XGBoost (p-value = 0.0003), DT (p-value = 0.0002), LGBM (p-value = 0.0154), RF (p-value = 0.0085), and GB (p-value = 0.0154). No significant difference was observed in the estimates between SVR and CatBoost (p-value = 0.1514). However, since the SVR model’s mean rRMSE and R² exceed that of the CatBoost, the SVR model was selected as the best model for predicting height-to-node ratio.

**Table 5:** *Post-hoc* results following the Friedman test, showing the average rank and Holm-adjusted one-sided Wilcoxon p-value of the models for predicting the height-to-node ratio. The model with the lower average rank value is the better model for prediction.

Model	Average ranks value	Comparison vs SVR	Holm-adjusted p-value
SVR	2.2000	–	–
CatBoost	2.2667	SVR vs CatBoost	0.1514
GB	3.5333	SVR vs GB	0.0154
RF	3.8000	SVR vs RF	0.0085
LGBM	4.3333	SVR vs LGBM	0.0154
XGBoost	5.2667	SVR vs XGBoost	0.0003
DT	6.6000	SVR vs DT	0.0002

### 3.5 Model testing (actual vs prediction)

**Figure 6:**
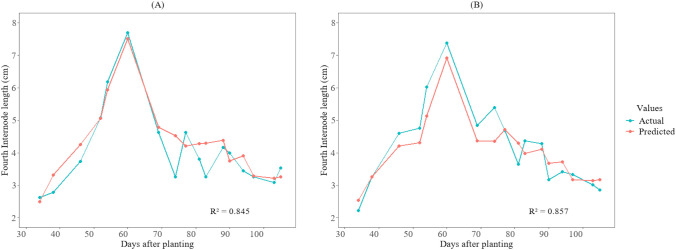
Growth variations of the fourth internode length between actual and predicted values for the cotton crops in the 2025 season at HortHill farm. **(A)** illustrates the actual vs predicted fourth internode length for plot 402, and **(B)** shows the actual vs predicted fourth internode length for plot 502.

**Figure 7:**
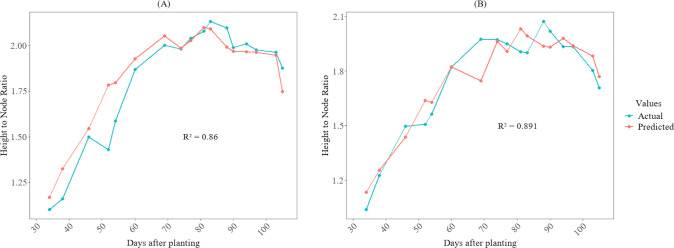
Growth variations of the height-to-node ratio between actual and predicted values for cotton crops in the 2025 season at HortHill farm. **(A)** illustrates the actual vs predicted height-to-node ratio for plot 402, and **(B)** shows the actual vs predicted height-to-node ratio for plot 502.

**Figures 6**, **7** illustrate the temporal pattern of the actual and predicted values for the two test plots (402 and 502). For predicting the fourth internode, the CatBoost model achieved an R^2^ of 0.845 for plot 402 and an R^2^ value of 0.857 for plot 502 for predicting fourth internode length, as shown in **Figure 6**. For the height-to-node ratio, the SVR model achieved R² of 0.86 when tested in plot 402 and R² value of 0.891 when tested in plot 502, as shown in **Figure 7**.

### 3.6 Relative importance of the vegetation indices for models’ prediction

**Figure 8:**
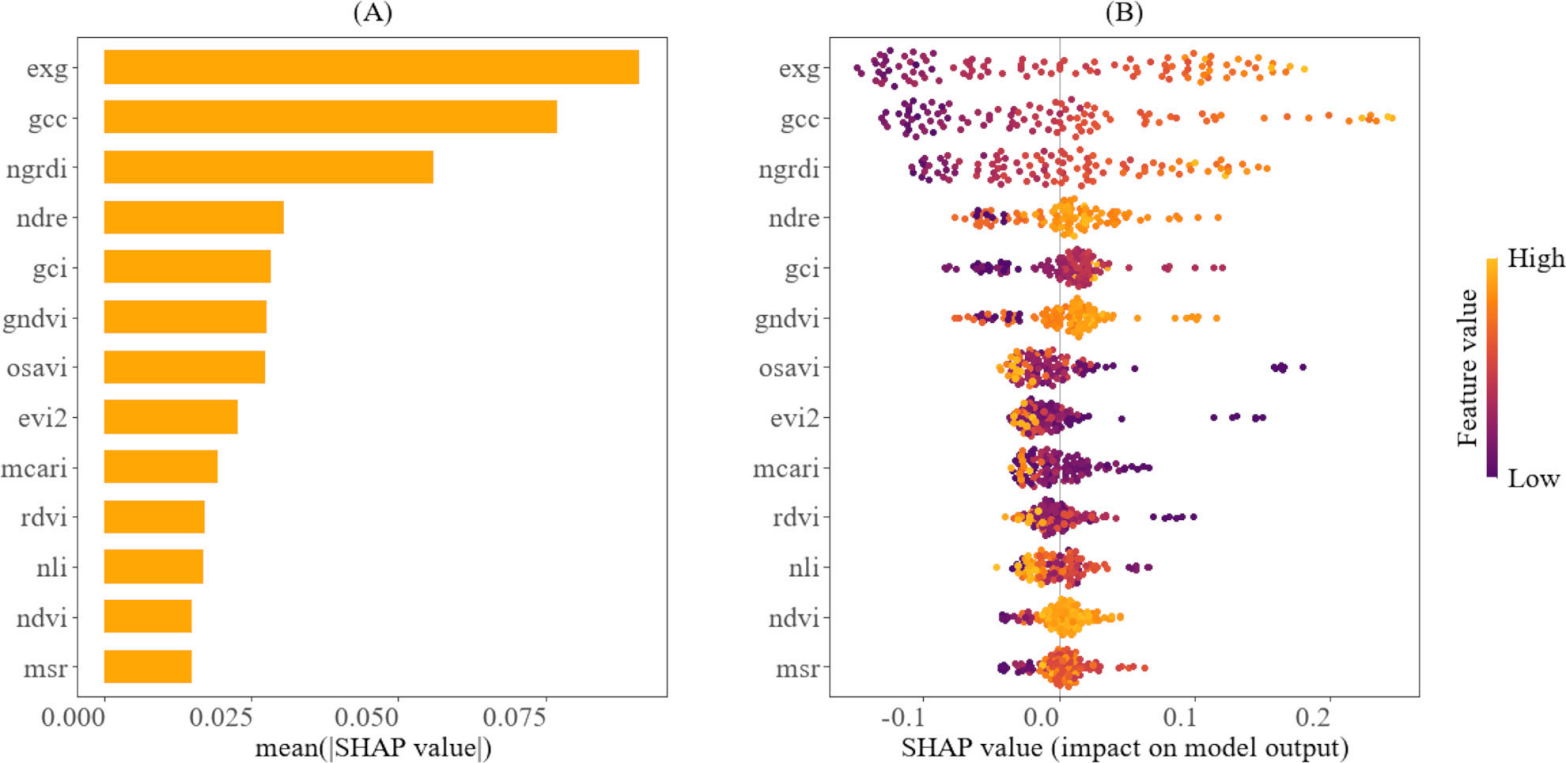
Explainability analysis of the CatBoost using SHAP for predicting the fourth internode length. **(A)** shows the variable ranking, indicating the importance of the vegetation indices to the model. **(B)** illustrates the relative importance of vegetation indices and their impact on model predictions, highlighting how their value distribution influences model predictions.

**Figure 9:**
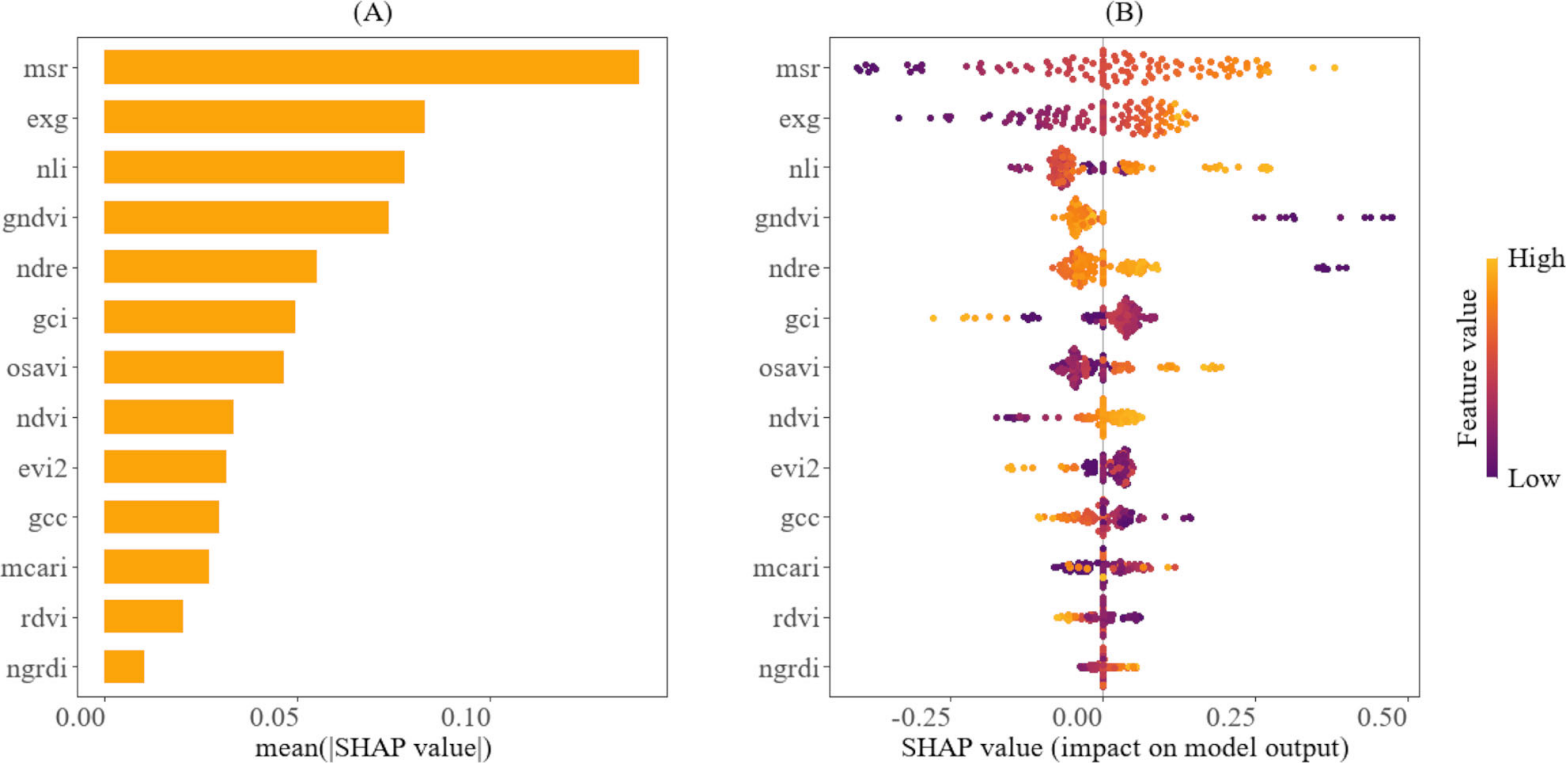
Explainability analysis of the SVR using SHAP for predicting the height-to-node ratio. **(A)** depicts the variable ranking, indicating the importance of the vegetation indices to the model. **(B)** illustrates the relative importance of vegetation indices and their impact on model predictions, highlighting how their value distribution influences model predictions.

The relative importance and the contributions of the predictor variables to the models, as interpreted through the SHAP method, are shown in **Figures 8**, **9**. According to **Figure 8A**, EXG, GCC, and NGRDI are ranked highest, indicating that they are the most significant variables in estimating the fourth internode length. Furthermore, **Figure 8B** illustrates that higher values (represented in yellow) of EXG, GCC, and NGRDI correspond to longer fourth internode lengths, while lower values (depicted in purple) are associated with shorter fourth internode lengths. This means that as the plant’s greenness increases, the model predicts a longer fourth internode length. Conversely, **Figure 8B** also reveals that for certain VIs, such as NLI and OSAVI, the relationship is reversed; higher values (shown in yellow) are associated with shorter fourth internode lengths, whereas lower values are associated with longer fourth internode lengths. This trend reveals the sensitivity of these indices to cotton canopy density and structure, which peaks in later growth stages amid denser canopies, when the elongation of the fourth internode is small.

In contrast, **Figure 9A** showcases that MSR, EXG, and NLI are the key variables for predicting the height-to-node ratio. **Figure 9B** also demonstrates that higher values of MSR, EXG, and NLI (indicated in yellow) are associated with a larger height-to-node ratio, while lower values (indicated in purple) correspond to a smaller height-to-node ratio. This pattern implies that higher values of these indices are associated with more vigorous vertical plant growth, while the lower values indicate less vigorous growth. It also reveals that some predictor variables, such as EVI2, GCI, and GCC, exhibit the opposite trend: higher values are associated with a smaller height-to-node ratio, while lower values are associated with a larger height-to-node ratio. The temporal trends for the top vegetation indices for predicting the fourth internode length and the height-to-node ratio are shown in the **Supplementary Material** in **Figures 1–4**.

## 4 Discussion

This study demonstrates that utilizing nonparametric, nonlinear machine learning algorithms in conjunction with vegetation indices derived from UAV imagery can accurately estimate two key canopy traits in cotton for PGR management. Such advancement allows for more precise and timely decisions regarding plant growth regulator applications. More broadly, our findings add to the growing evidence that integrating ML with UAV data offers a more accurate and non-destructive way to assess spatial variability in cotton growth (Ashapure et al., 2020; Ma et al., 2021).

Among the ML regression algorithms evaluated, SVR and CatBoost emerged as the top-performers for estimating the height-to-node ratio and the fourth internode length, respectively. As illustrated in **Figure 5B**, the height-to-node ratio exhibits a relatively smooth, gradually increasing quasi-monotonic trend throughout the season, with moderate variation across plots. This pattern aligns well with SVR’s capability to fit a smooth, nonlinear function, effectively balancing model complexity and error tolerance (Schölkopf and Smola, 2002). In contrast, **Figure 5A** shows that the fourth internode length increases to its highest values around mid-season, then declines rapidly later in the season, reflecting a more complex, non-monotonic response to the VIs. CatBoost uses ordered boosting and ordered target statistics to mitigate prediction shifts caused by target leakage, thereby enhancing generalization and reducing overfitting (Prokhorenkova et al., 2018). These features likely contributed to its superior performance in predicting the fourth internode length. On the other hand, DT performed the worst in both prediction scenarios. This outcome is consistent with the well-known limitations of CART-style regression trees, which split the feature space using simple, axis-aligned partitions and fit piecewise-constant prediction functions over the resulting regions (Hastie et al., 2009). These limitations may have hindered the single trees’ ability to capture smooth, nonlinear, and multivariate relationships between vegetation indices and cotton traits. Moreover, the differing top-performing algorithms for these traits suggest that the suitability of a particular algorithm depends on the specific trait and its underlying structure in relation to the VIs. Consequently, different traits may exhibit varying levels of complexity, making certain algorithms better suited to capture these complexities than others. This trait-specific variability is consistent with prior studies; for instance, Flynn et al. (2024) reported that SVR was the best model for predicting cotton height and node, whereas RF was the best model for predicting cotton leaf area index. Similarly, Han et al. (2023) also reported that the LGBM was a better model for predicting the net photosynthetic rate, while the RF was a better model for predicting the fraction of absorbed photosynthetically active radiation. Singhal et al. (2024) found that Kernel ridge regression outperforms RF and SVM when estimating leaf chlorophyll in maize plants using multispectral UAV images. Additionally, Upreti et al. (2019) reported that least-squares linear regression was the most effective for leaf area index prediction, while RF was superior for canopy chlorophyll content prediction in wheat when estimating these parameters from Sentinel-2 data. Collectively, these studies emphasize that no one ML algorithm consistently outperforms others across all prediction tasks.

Furthermore, the SHAP analysis results indicate that certain variables have a greater influence on the model’s predictions than others. For example, MSR, EXG, and NLI were identified as the most significant contributing indices for predicting the height-to-node ratio, whereas EXG, GCC, and NGRDI were the most significant contributing indices for predicting the fourth internode length. This variation in feature importance offers practical guidance for selecting the most suitable UAV sensor when monitoring these traits separately, thereby optimizing hardware and data processing costs. For example, given that the RGB VIS features are the most influential in estimating the fourth internode length, a cost-effective RGB sensor would be adequate for monitoring this trait. In contrast, the reliance on NIR-derived indices for estimating the height-to-node ratio justifies the investment in a multispectral sensor. Furthermore, these insights enable precise feature engineering by prioritizing the most informative indices, thereby reducing model redundancy and complexity. Additionally, the prominence of the MSR and NLI in predicting the height-to-node ratio is not surprising. These indices are derived from NIR and red bands and are strongly correlated with cotton growth parameters, such as biomass, leaf area index, plant height, node number, and yield (Ma et al., 2021; Lacerda et al., 2025). This strong correlation arises from their reduced sensitivity to the canopy optical and geometrical properties (Chen, 1996), making them ideal for measuring integrated structural traits, such as the height-to-node ratio. Conversely, GCC and NGRDI, which quantify the greenness of a plant, are also strongly correlated with chlorophyll content in the plant chloroplasts (Seyednasrollah et al., 2021; Pazhanivelan et al., 2023). Chlorophyll absorbs light for photosynthesis, generating energy that supports cell expansion and stem elongation, thereby contributing to the elongation of the fourth internode length. Interestingly, EXG, an RGB-based index, emerged as one of the most influential predictors for both traits. Previous studies have shown that EXG is strongly correlated with LAI (Wang et al., 2025) and the vertical growth of plants (Psiroukis et al., 2023), highlighting its sensitivity to overall plant architecture and canopy greenness, which aligns with the traits estimated in this study.

The developed models accurately captured the temporal trends of the measured and predicted values for both the fourth internode length and the height-to-node ratio, as depicted in **Figure 6** and **Figure 7**. This demonstrates their effectiveness in monitoring the dynamic growth of cotton plants. This capability is particularly vital because cotton, which has an indeterminate growth habit, often produces excessive vegetative growth that can lead to dense canopies. Such conditions, if left unchecked, may result in fruit shedding and ultimately yield loss (Collins et al., 2017). However, despite the overall strong performance, small discrepancies between observed and predicted values are visible on some sampling dates (**Figures 6**, **7**). These deviations are likely due to inherent uncertainties in ground-truth measurements, as well as residual variations in canopy reflectance (Wang et al., 2023) and micro-shading (Yin et al., 2024) that may persist even after radiometric calibration. Nevertheless, the models reliably estimate the growth patterns of these traits, enabling rapid identification of excessive growth in the field for timing PGR application. This approach substantially reduces the need for manual monitoring of these traits, increases the identification of spatial variability, and thereby enhances data-driven management in cotton production.

While this study has its strengths, it is important to recognize several limitations. The models were trained on data from a single cotton cultivar over one growing season, with a limited sample size. Future studies should incorporate multi-season, multi-site datasets that include a wider range of cultivars and environmental conditions, as well as larger sample sizes. Such an expanded dataset will enhance the robustness and generalizability of the models, leading to improved predictive accuracy and better monitoring of cotton growth. This, in turn, will facilitate better timing recommendations for plant growth regulator (PGR) applications.

## 5 Conclusion

This study has demonstrated that nonparametric, nonlinear ML algorithms can accurately predict the cotton fourth internode length and height-to-node ratio from VIs. Comparative evaluation among these models revealed that CatBoost consistently outperforms other models in predicting fourth internode length, while SVR demonstrated a superior performance in estimating the height-to-node ratio. The use of these models, which utilize Vis to estimate these traits, represent a significant advancement in cotton monitoring these traits. This approach can greatly alleviate the challenges associated with manual measurements, thereby optimizing the timing of PGR applications.

## Data Availability

The raw data supporting the conclusions of this article will be made available by the authors, without undue reservation.
