# Supplementary material for: In situ estimation of cotton fourth internode length and height-to-node ratio using UAV-derived vegetation indices and machine learning algorithms
Source: Front Plant Sci. 2025 Dec 16;16:1722440. doi: 10.3389/fpls.2025.1722440 (PMC12748212; doi:10.3389/fpls.2025.1722440)
Supplement: Supplementary file 1 [file DataSheet1.pdf]

Supplementary Material

1 Supplementary Figures and Tables

1.1 Supplementary Figures

Supplementary Figure 1. The maps of the modified simple ratio (MSR) index of plot 402 from 32 to 105 days after planting (DAP). Yellow indicates low MSR, and dark blue indicates high MSR values. MSR is the most influential vegetation index for predicting the height-to-node ratio.

Supplementary Figure 2. The maps of the Excessive Green (EXG) index of plot 402 from 32 to 105 days after planting (DAP). Yellow indicates low EXG, and dark blue indicates high EXG values. EXG is the most influential vegetation index for predicting the fourth internode length.

Supplementary Figure 3. The maps of the modified simple ratio (MSR) index of plot 502 from 32 to 105 days after planting (DAP). Yellow indicates low MSR, and dark blue indicates high MSR values. MSR is the most influential vegetation index for predicting the height-to-node ratio.

Supplementary Figure 4. The maps of the Excessive Green (EXG) index of plot 502 from 32 to 105 days after planting (DAP). Yellow indicates low EXG, and dark blue indicates high EXG values. EXG is the most influential vegetation index for predicting the fourth internode length.

1.2 Supplementary Table

Supplementary Table 1. Description of the hyperparameters used during model training by means of the grid search tuning technique

Model	Hyperparameters	Values
Support Vector Regression (SVR)	kernel	['rbf','sigmoid','poly']
	c	[0.01, 100, prior = 'log-uniform']
	gamma	['scale', 'auto']
	coef	[0.0, 1.0]
	epsion	[0.001, 0.5, prior='log-uniform']
Decision Tree (DT)	max_depth	[3, 6]

	min_samples_split	[2, 10]
	min_samples_leaf	[2, 5]
	max_features	['sqrt', 'log2', None, 0.8]
	ccp_alpha	[0.0, 0.01]
Gradient Boosting (GB)	n_estimators	[200, 800]
	learning_rate	[0.02, 0.1, prior='log-uniform']
	max_depth	[2, 4]
	subsample	[0.9, 1.0]
	min_samples_split	[2,10]
	min_sample_leaf	[1, 4]
	max_features	['sqrt', 'log2', None, 0.8]
Random Forest (RF)	n_estimators	[200, 800]
	max_depth	[4, 12]
	min_samples_split	[2, 10]
	min_sample_leaf	[1, 4]
	max_features	['sqrt', 'log2', 0.5]
	bootstrap	[True]
	max_samples	[None, 0.7, 0.9]
eXtreme Gradient Boosting (XGBoost)	n_estimators	[300, 600]
	learning_rate	[0.01, 0.2, prior='log-uniform']

	max_depth	[3, 10]
	min_child_weight	[3, 10]
	gamma	[0.0, 5.0]
	subsamples	[0.5, 1.0]
	colsample_bytree	[0.6, 0.8, 1.0]
	reg_alpha	[0.0, 1.0]
	reg_lambda	[0.01, 50.0, prior='log-uniform']
Categorical Boosting (CatBoost)	depth	[4, 8]
	learning_rate	[0.001, 0.3, prior='log-uniform']
	n_estimators	[300, 1500]
	l2_leaf_reg	[0.01, 10]
	bootstrap_type	['Bayesian']
	bagging_temperature	[0, 5]
Light Gradient Boosting Machine (LightGBM)	n_estimators	[300, 900]
	learning_rate	[0.03, 0.1, prior = 'log-uniform']
	max_depth	[5, 10]
	num_leaves	[15, 31]
	min_child_samples	[1, 10]
	min_split_gain	[0.0, 0.01]

	subsample	[0.5, 1.0]
	subsample_freq	[0, 1]
	colsample_bytree	[0.5, 1.0]
	reg_alpha	[0.0, 1.0]
	reg_lambda	[0.001, 5.0, prior='log-uniform']
