# Supplementary figures and images for: In situ estimation of cotton fourth internode length and height-to-node ratio using UAV-derived vegetation indices and machine learning algorithms
Source: Front Plant Sci. 2025 Dec 16;16:1722440. doi: 10.3389/fpls.2025.1722440 (PMC12748212; doi:10.3389/fpls.2025.1722440)

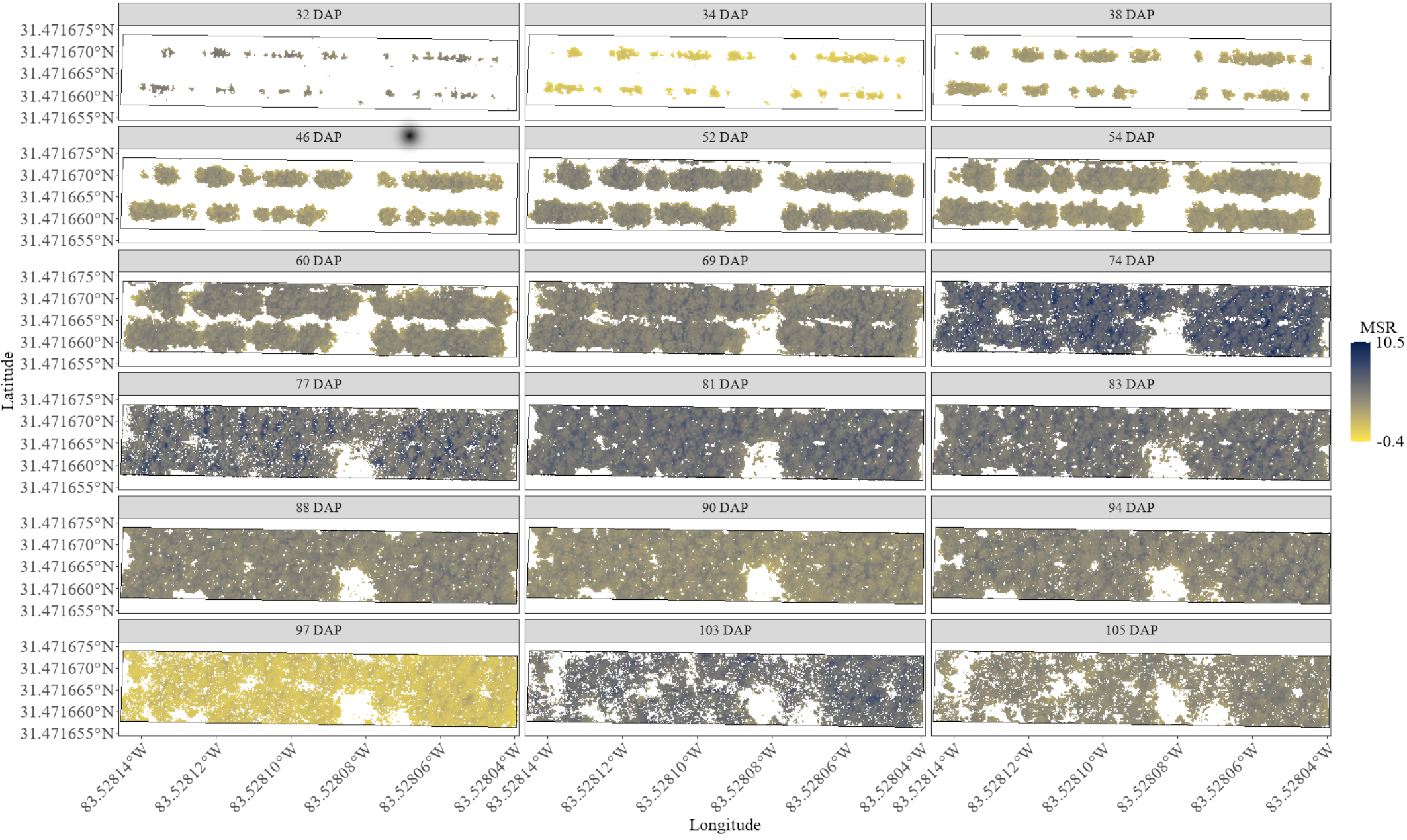

Supplement: Supplementary file 2 [file Image1.tif]

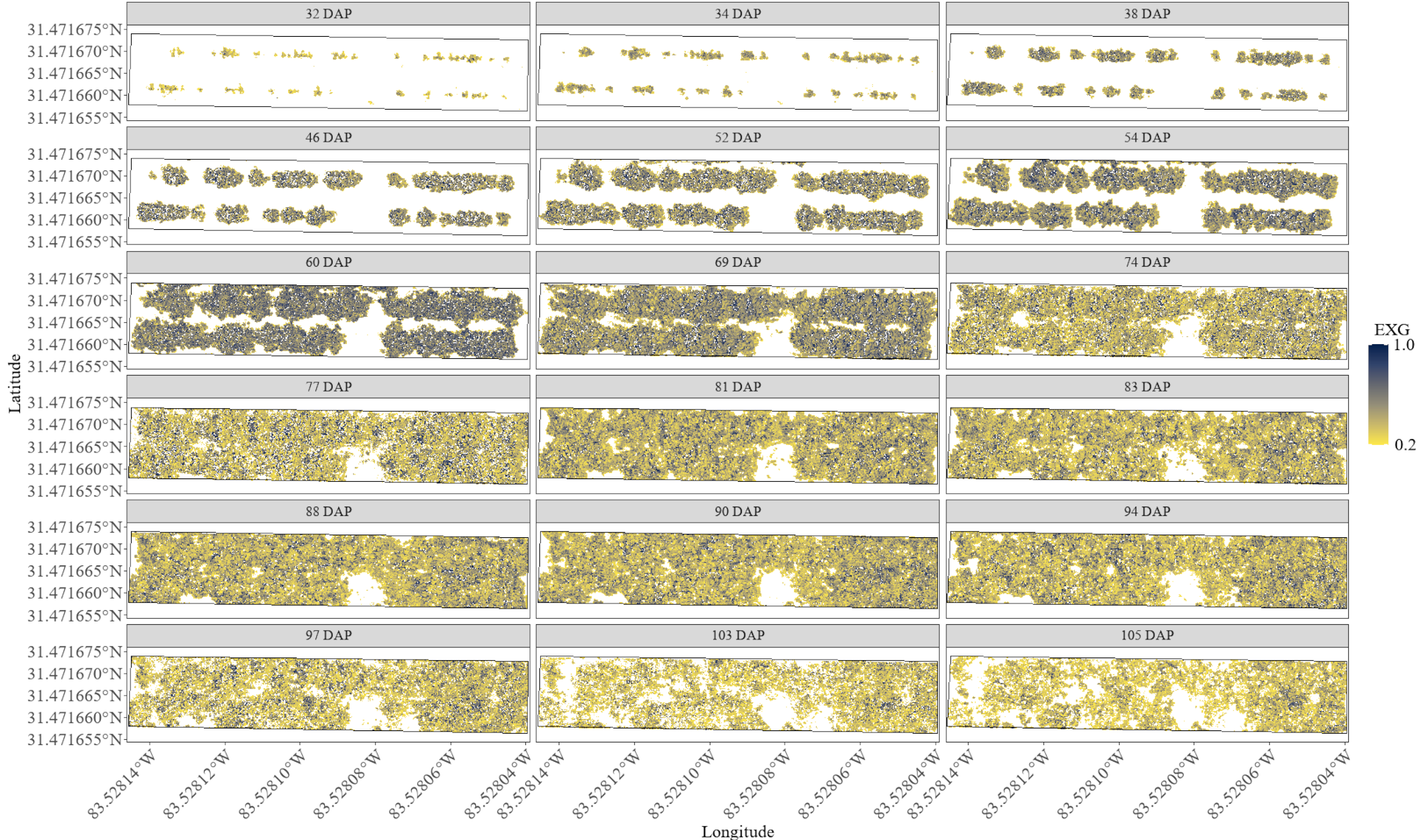

Supplement: Supplementary file 3 [file Image2.tif]

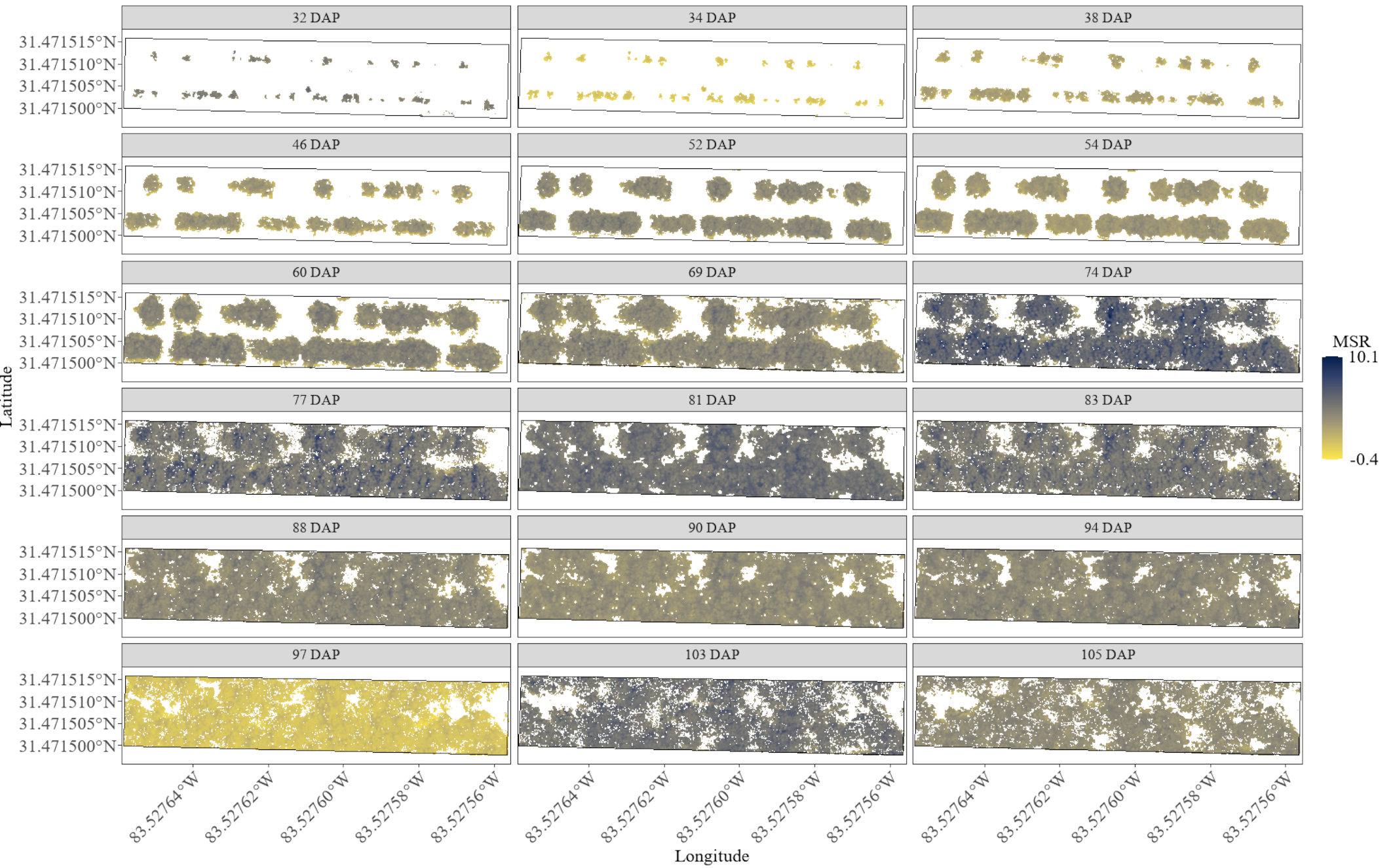

Supplement: Supplementary file 4 [file Image3.tif]

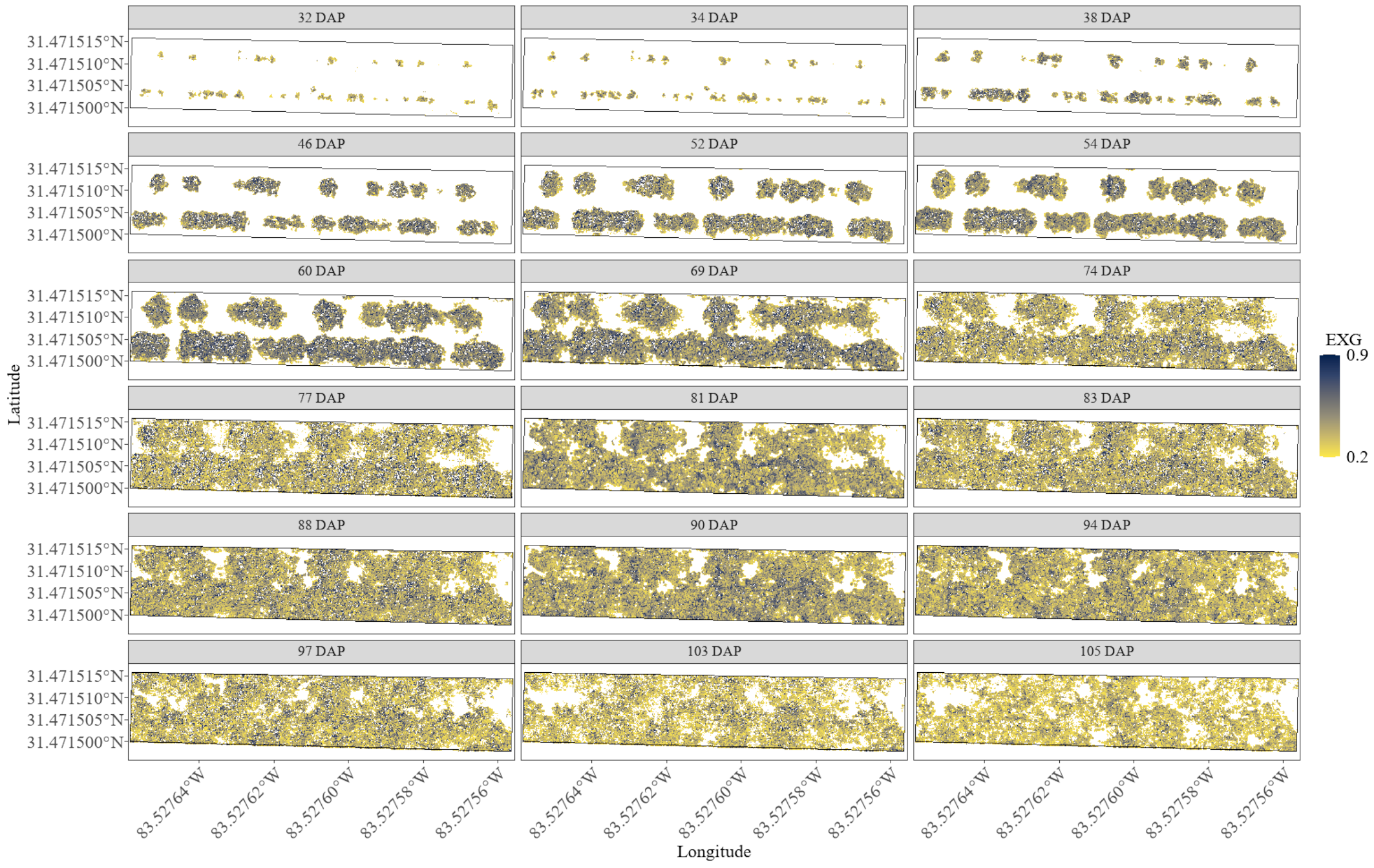

Supplement: Supplementary file 5 [file Image4.tif]
